# Spontaneous Regression of Merkel Cell Carcinoma of the Male Breast with Ongoing Immune Response

**DOI:** 10.7759/cureus.3589

**Published:** 2018-11-14

**Authors:** Yugmel Nijjar, Gilbert Bigras, Patricia Tai, Kurian Joseph

**Affiliations:** 1 Miscellaneous, Cumming School of Medicine, University of Calgary, Calgary, CAN; 2 Pathology, Cross Cancer Institute, University of Alberta, Edmonton, CAN; 3 Radiation Oncology, Allan Blair Cancer Center, University of Saskatchewan, Regina, CAN; 4 Radiation Oncology, Cross Cancer Institute, University of Alberta, Edmonton, CAN

**Keywords:** spontaneous regression, merkel cell carcinoma, immune response

## Abstract

Merkel cell carcinoma (MCC) is a rare cutaneous neuroendocrine tumor arising predominantly on sun-exposed skin among the elderly. The most common location is the head and neck, followed by the extremities. MCCs are highly aggressive tumors and rarely undergo spontaneous regression. We report a case of MCC which presented as a painless breast lump in an elderly male where the tumor regressed spontaneously after a biopsy.

## Introduction

Merkel cell carcinoma (MCC) is a rare cutaneous neuroendocrine tumor arising from the dermoepidermal junction of the skin and has features of both epithelial and neuroendocrine origin [1-2]. MCC often presents as a painless red-violet nodule on ultraviolet-exposed skin [3]. The tumor is extremely aggressive and locoregional spread or metastasis is common at initial presentation. However, MCC rarely regresses spontaneously. We report a case of MCC in an elderly gentleman that presented with a painless breast lump and underwent spontaneous regression (SR) following core biopsy.

## Case presentation

**Figure 1:**
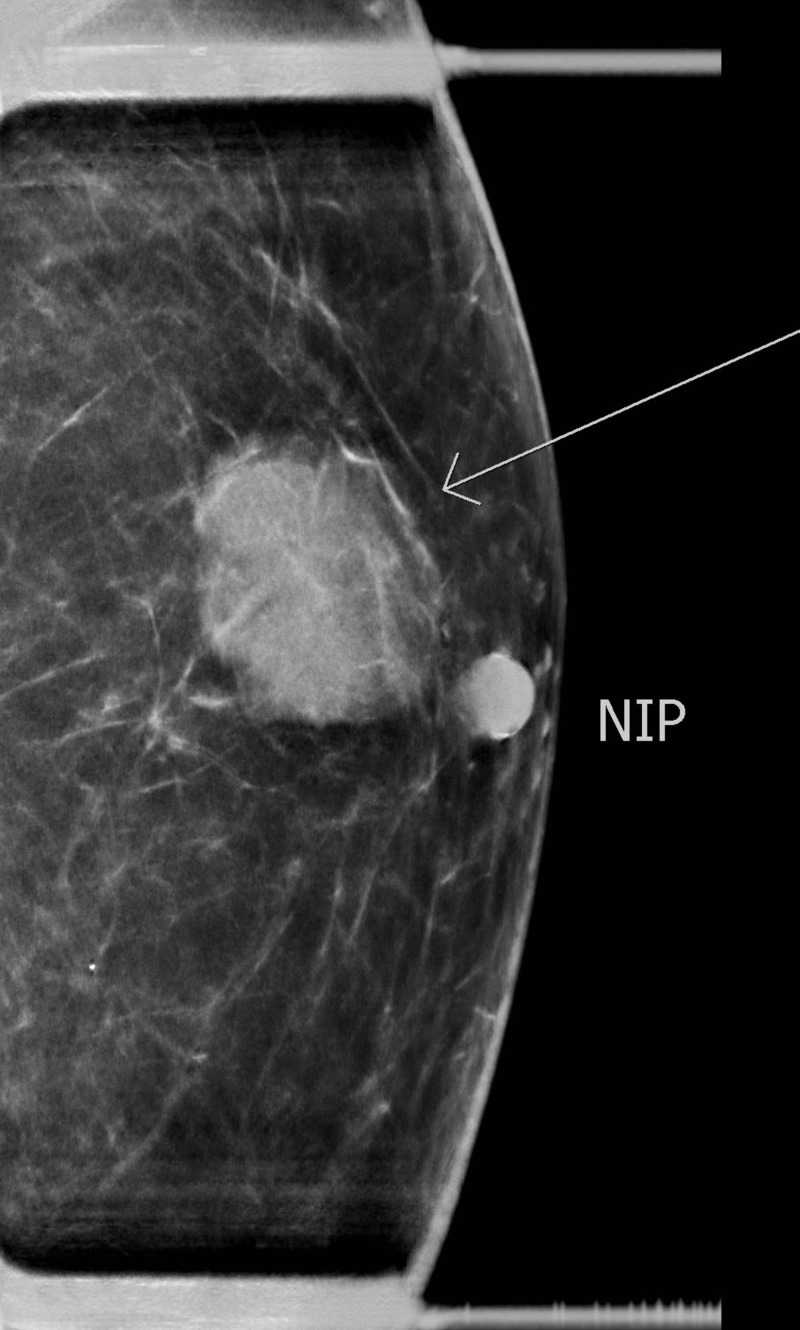
Mammogram of the left breast showing an irregular soft tissue mass at 12-o’clock measuring 2.4 x 1.4 cm

**Figure 2:**
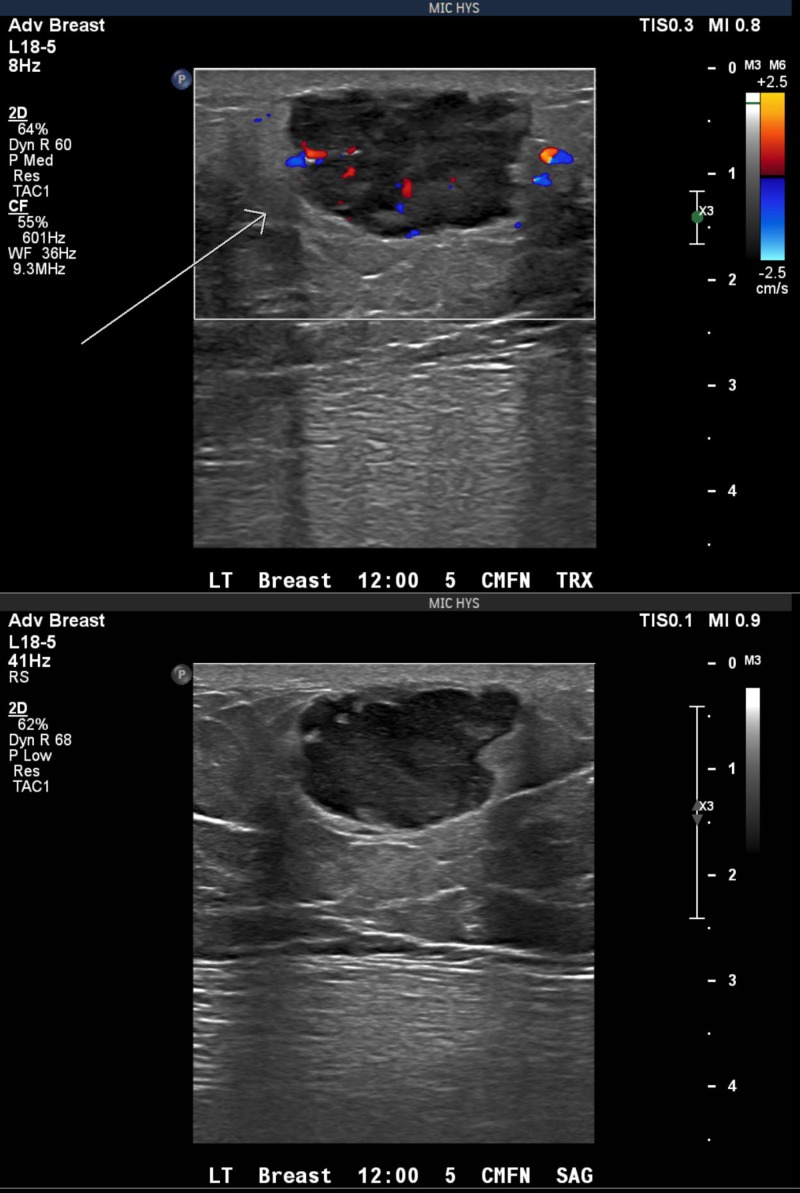
Ultrasound of the left breast showing increased vascularity

**Figure 3:**
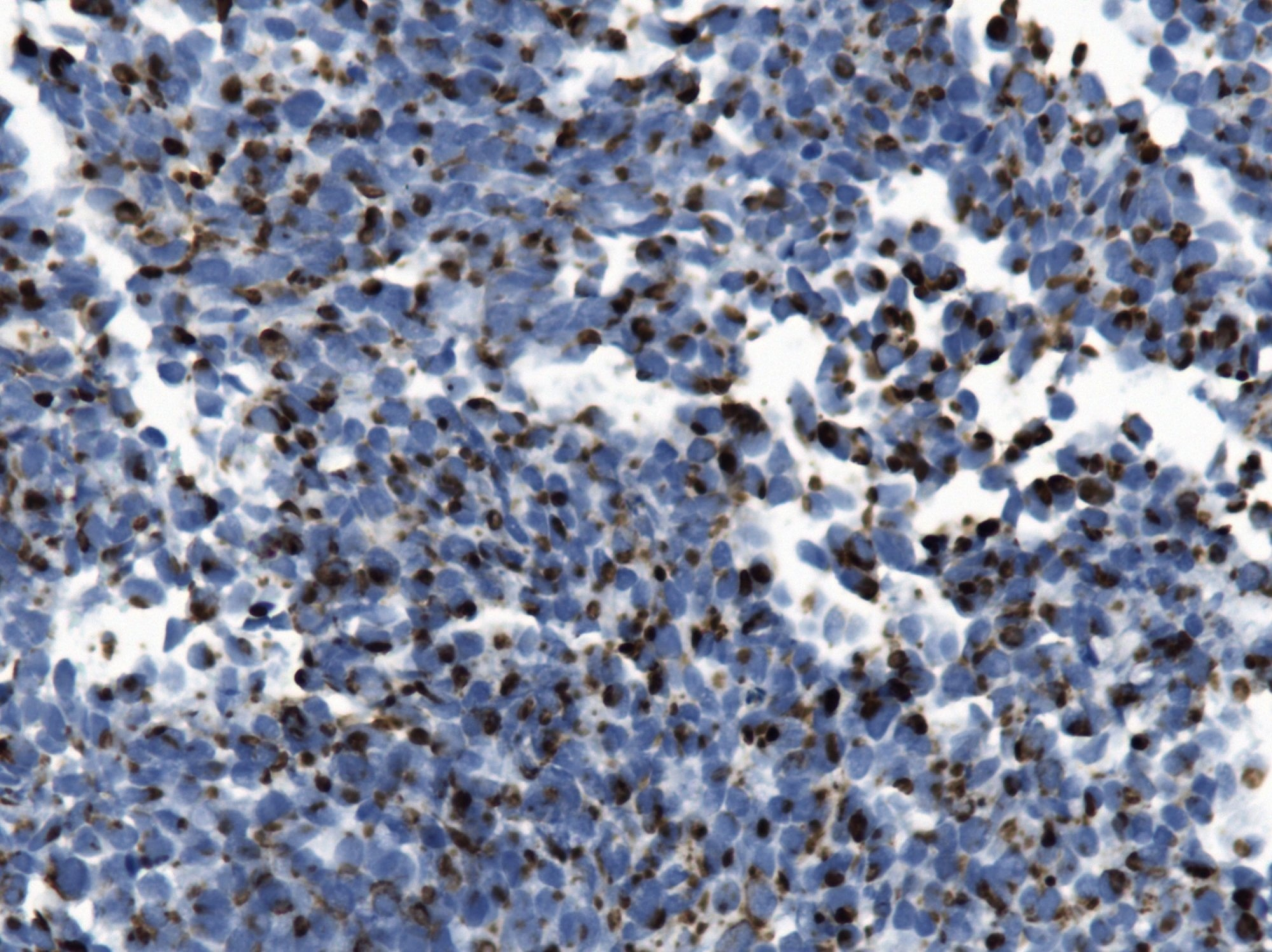
Cytokeratin 20 shows typical dot-like cytoplasmic staining

**Figure 4:**
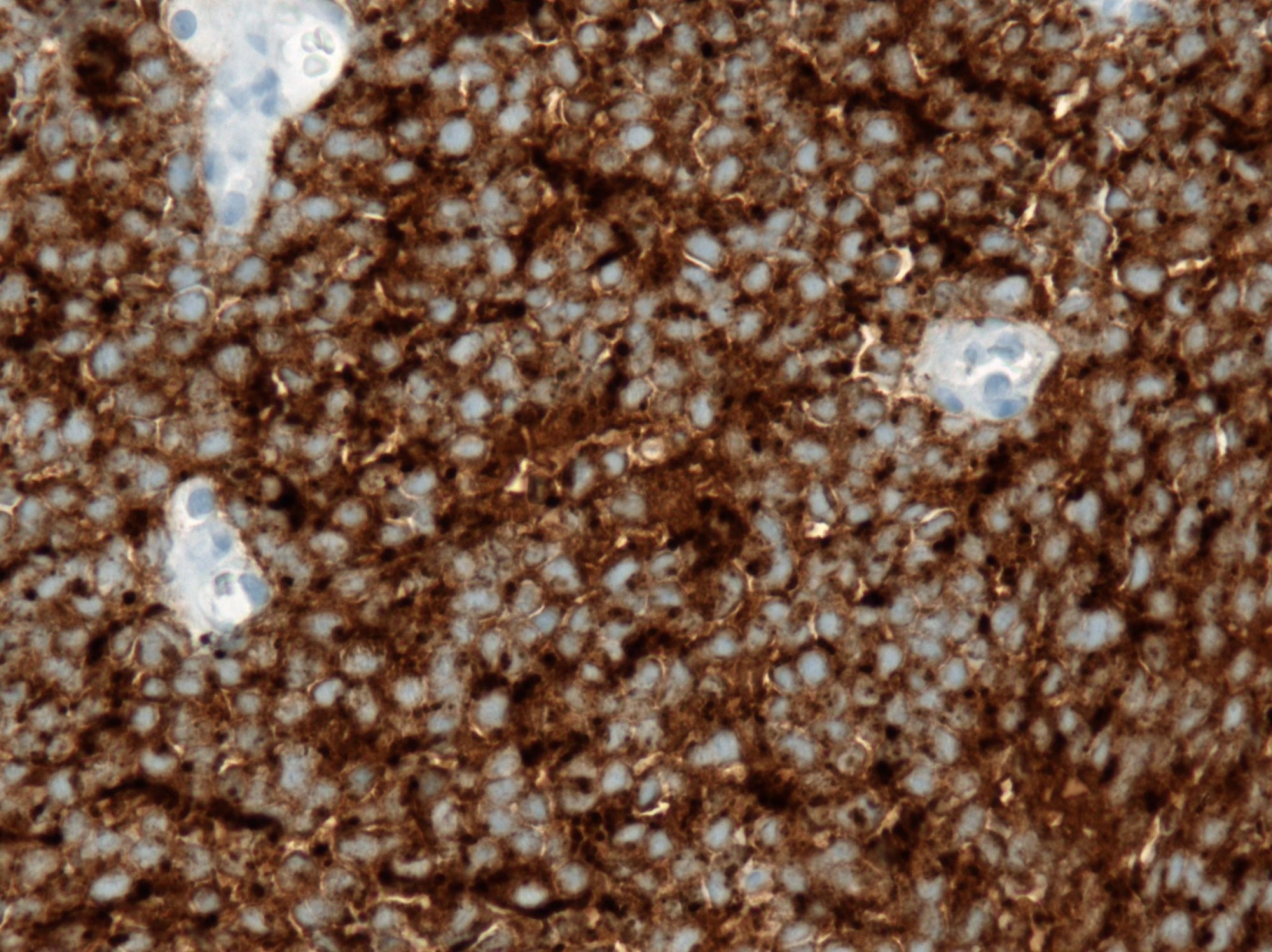
Abundant granular, diffuse, and strong cytoplasmic staining illustrating the neuroendocrine differentiation (synaptophysin)

A 77-year-old gentleman presented with a painless left breast lump of six months duration. The lesion was subcutaneous and skin was intact without any colour change. The patient underwent a mammogram (Figure 1) and an ultrasound (Figure 2), which revealed an irregular soft tissue mass at the 12-o’clock position, measuring 2.4 x 1.4 cm (T2). There was no calcification but an increased vascularity was noted. No lymph node was palpable or detected by imaging. Subsequently, the patient underwent a core biopsy that revealed sheets of poorly differentiated malignant small blue cells at a high mitotic rate, focally demonstrating rhabdoid-type features. AE1/AE3, neuron-specific enolase, and cytokeratin 20 (CK20) showed typical strong cytoplasmic dot positivity (Figure 3). Neuroendocrine markers (synaptophysin and neural cell adhesion molecule (CD56)) and B-cell lymphoma 2 (BCL-2) were also positive, as was cytoplasmic positivity for beta-catenin (Figure 4). S100, cytokeratin 5 (CK5), thyroid transcription factor 1 (TTF1), napsin A, GATA3, estrogen receptors (ER), progesterone receptors (PR), and human epidermal growth factor receptor-2 (HER-2)/neu protein were all negative. The immunohistochemical profile and pattern of cytokeratin staining were most in keeping with MCC. Retrospective pathological workup showed the MCC tumor was negative for polyomavirus, and a small amount of tumor infiltrating lymphocytes (TILs) was noted with a cluster of differentiation 3 (CD3) immunoassay. An 18F-fluorodeoxyglucose positron emission tomography/computed tomography (^18^FDG PET/CT) performed two weeks after biopsy did not show any FDG-avid lesion, except for a nonspecific uptake in multiple mediastinal lymph nodes. The tumor was staged as IIA (T2N0). His scheduled lumpectomy and sentinel lymph node biopsy were canceled since the PET imaging was negative and there was no mass felt on clinical evaluation. A follow-up CT at three months was also normal without any abnormality at the site of the lump. ^18^FDG PET/CT repeated at nine months showed hypermetabolic activity in multiple lymph node regions and in the spleen. The findings were considered as reactive as opposed to metastatic disease since biopsy of the supraclavicular node demonstrated reactive changes only. The patient is on continued follow-up up to three years to rule out local recurrence or metastatic disease in the future.

## Discussion

MCC was first described by Toker in 1972 as a trabecular carcinoma of the skin [4]. MCC often presents as a painless red-violet nodule on sun-exposed skin among the elderly. MCC occurs most commonly on the skin of the head and neck, followed by the extremities [5]. MCC presenting as a breast lump is rare.

MCC is highly aggressive and commonly presents with locally advanced or metastatic disease. However, spontaneous regression (SR) occurs rarely. The estimated incidence is 1.5% - 3% but may be greater than reported [6-7]. The mechanism of SR is not clearly understood. Most instances of SR occurred following a diagnostic biopsy or incomplete excision of the primary tumor [8]. There is also evidence of increased immune activity around the tumor regression site in the form of tumor infiltrating lymphocytes [9]. Several studies reported heavy infiltration of CD4+, CD8+, CD3+ T lymphocytes, and foamy macrophages around the tumor nests [6, 10]. These findings suggest the role of T cell-mediated immune response in the development of tumor regression resulting in apoptosis and cellular necrosis [11]. In our case, only minimal infiltrating TILs were seen. However, the mild and persistent reactive changes seen in the lymph nodes 10 months post-SR may represent continued T cell-mediated immune response. Considering the ongoing immune response, the patient will be closely monitored for local recurrence or metastatic disease. 

MCC has shown to be an immunologically responsive disease and immune checkpoint inhibitors are effective for advanced MCC. Pembrolizumab is the first immune checkpoint inhibitor showing objective tumor regression in a patient with MCC.

## Conclusions

Our report further supports the observation that spontaneous regression in MCC can occur after the diagnostic biopsy. As T cell-mediated immune response is a key player in the regression of MCC, further research should seek the possibility of developing immune modulation as a method of treating MCC. MCC has shown to be an immunologically responsive disease and immune checkpoint inhibitors are effective for advanced MCC.
